# The Production of Somatostatin Interneurons in the Olfactory Bulb Is Regulated by the Transcription Factor Sp8

**DOI:** 10.1371/journal.pone.0070049

**Published:** 2013-07-23

**Authors:** Xuhua Jiang, Mingguang Zhang, Yan You, Fang Liu

**Affiliations:** 1 Institutes of Brain Science and State Key Laboratory of Medical Neurobiology, Fudan University, Shanghai, China; 2 Department of Neurosurgery, Huashan Hospital, Fudan University, Shanghai, China; Baylor College of Medicine, United States of America

## Abstract

Somatostatin (Som), one of the most concentrated neuropeptides in the brain, is highly expressed in the olfactory bulb (OB). However, the temporal profile by which OB somatostatin-expressing (Som+) interneurons are produced and the molecular mechanisms controlling this profile are totally unknown. In the present study, we found that all the Som+ interneurons in the mouse external plexiform layer (EPL) and the rat glomerular layer (GL) express the transcription factor Sp8.Using the 5-bromo-2′-deoxyuridine (BrdU) birth dating method, combined with immunostaining, we showed that the generation of Som+ interneurons in the mouse and rat OB is confined to the later embryonic and earlier postnatal stages. Within the mouse OB, the production of Som+ interneurons is maximal during late embryogenesis and decreases after birth, whereas the generation of Som+ interneurons is low during embryogenesis and increases gradually after birth in the rat OB. Interestingly, genetic ablation of Sp8 by cre/*loxP*-based recombination severely reduces the number of Som+ interneurons in the EPL of the mouse OB. Taken together, these results suggest that Sp8 is required for the normal production of Som+ interneurons in the EPL of the mouse OB.

## Introduction

The olfactory bulb is one of the two regions in the brain where neurogenesis persists throughout life. Migratory neuroblasts generated by the subventricular zone (SVZ) progenitors travel along the rostral migratory stream (RMS) to the olfactory bulb and become local interneurons [1], [2]. The interneurons in the olfactory bulb are mainly located in either the granular cell layer (GCL), the external plexiform layer (EPL), or the glomerular layer (GL). Based on the specific expression of classical neurochemical markers, the OB interneurons can be classified into several types, such as calretinin (CR), calbindin (CB), tyrosine hydroxylase (TH) and parvalbumin (PV) [3]–[6].

Somatostatin is a regulatory neuropeptide mainly concentrated in local GABAergic interneurons restricted to the EPL in mouse OB [7] and GL in rat OB [8]. Using the BrdU pulse-labeling method, we have shown that the neurons in the EPL are mainly generated around birth [6], [9]. However, the timing of the generation of somatostatin interneurons in the OB remains largely unknown.

Both intrinsic and extrinsic mechanisms are involved in the regulation of OB neurogenesis. Previous studies have shown that both the specification and differentiation of the dopaminergic TH+ cells are regulated by Pax6 [10], [11]. We have shown that many PV+ cells in the EPL of the rat OB originate from the postnatal SVZ [6] and are regulated by the transcription factor Sp8 [9]. However, the molecular mechanisms that regulate the production of Som+ cells in the EPL of the OB are unclear.

In this study, we demonstrated that Sp8 was expressed in Som+ interneurons in both the mouse EPL and the rat GL. Using BrdU birth dating analysis, we found that Som+ interneurons in the mouse and rat OB are generated during different developmental windows. More importantly, and the density of Som+ cells in the EPL of the mouse OB was significantly reduced by genetic ablation of Sp8. These results indicate that *Sp8* is required for the production and/or survival of the Som+ interneurons in the OB.

## Results

### Som+ Cells Express Sp8 in the OB of Mice and Rats

Previous studies have shown that Sp8, a member of the Sp1 zinc finger transcription factors, is expressed by fully differentiated interneurons in the mouse and rat OB [9], [12], [13]. However, whether the Som+ cells in the adult rat and mouse OB express Sp8 has not been investigated.

**Figure 1:**
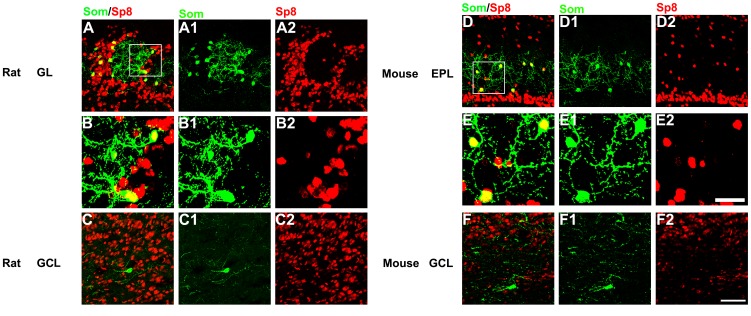
Virtually all of the Som+ cells express Sp8 in the OB. (A) The Som+ cells in the GL of the adult rat OB express *Sp8.* (B) High magnification of the boxed areas in A showing the Som+/Sp8 cells. (C) The Som+ cells with a larger soma in the GCL do not express Sp8 in the rat OB. (D) The Som+ cells in the EPL of the mouse OB express Sp8. (E) High magnification of the boxed areas in D showing the Som+/Sp8+ cells. (F) The Som+ cells with a larger soma in the GCL do not express Sp8 in the mouse OB. Scale bars: 50 µm (in F2 applies to A, C, D and F), 20 µm (in E2” applies to B and E).

In accordance with a previous study [8], our results showed that Som+ cells are mainly located in the GL of the rat OB. The Som+ cells distributed in the GL layer did not colocalize with CR, CB or TH [8] (Fig. S1 A, B and C). More importantly, majority of the Som+ cells expressed Sp8 (Fig. 1 A and B), whereas, the Som+ cells in the GCL does not (Fig. 1C). The mouse is becoming more and more widely used as a model of nervous system function. However, as neuroanatomical divergence in the OB exists between the mouse and the rat [14], we also explored the expression of Sp8 in the Som+ cells in the mouse OB. Consistent with other studies, our results showed that the Som+ cells were mainly located in the inner EPL, and a few were scattered in the GCL of the adult mice [7] (Fig. 1 D–F). We found that the majority of the Som+ cells in the EPL expressed Sp8 (Fig. 1 D), whereas the Som+ cells in the GCL also did not express Sp8 (Fig. 1 F). These results suggested that two different subgroups of Som+ cells (Sp8*+ and* Sp8*−*) are likely to exist in the OB.

### Somatostatin-expressing Interneurons in the OB are Produced Around Birth

**Figure 2:**
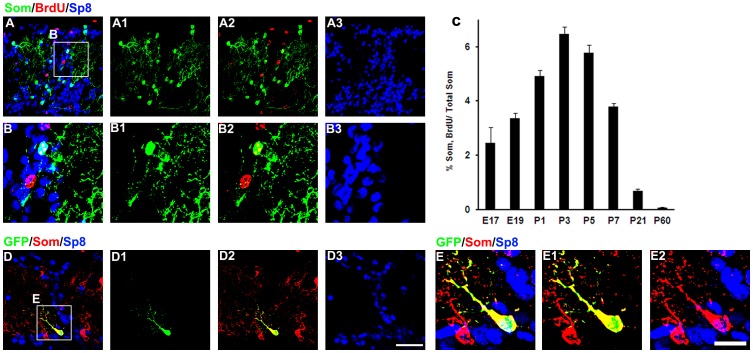
Som+ cells in the GL of rat OB are produced after birth and express Sp8. (A) Representative images of the BrdU+/Som+/Sp8+ cells in the GL of the rat OB. (B) High magnification of the boxed areas in A showing the BrdU+/Som+/Sp8+ cells. (C) Quantification of the percentage of Som+ cells that were labeled with BrdU in the GL of the rat OB. (D) Representative images of the GFP+/Som+/Sp8+ cells in the GL of rat OB four weeks after the replication-incompetent retroviruses encoding GFP were injected into the SVZ of the P0 rat. Scale bars: 50 µm (in D3 applies to A and D), 20 µm (in E2 applies to B and E).

The generation of new neurons in the OB begins in the embryonic stages and continues throughout life. BrdU, an analog of thymidine, can replace thymidine and permanently and incorporate into the newly synthesized DNA of dividing cells during the S-phase of the cell cycle. To investigate the time course of the interneuron production in the GL of rat OB, a single injection of BrdU was administered into rats at different time points (see details in “Materials and Methods”). Six to seven weeks after the injection, the majority of the BrdU-labeled Som+ interneurons migrated into the OB and acquired the appropriate phenotype and laminar position [9]. Colocalization of BrdU with Som was found in the GL, and majority of these double labeled cells express Sp8 (BrdU+/Som+/Sp8+, Fig. 2 A and B). The proportion of Som+ cells that were labeled with BrdU at each time point in the GL was analyzed, and a “bell-like” temporal production pattern was observed (Fig. 2 C). Since the Som+ cells in the GCL of rat and mouse did not express Sp8, we did not evaluated the generation of them in the present study. The proportion of newly generated Som+ cells (BrdU+/Som+) reached a peak at P3 (6.47±1.82%, n = 3) and declined slowly. No BrdU+/Som+ cells were found when BrdU was used to treat P60 rats, suggesting that the Som+ cells are not generated in the adult rats.

We used an oncoretrovirus-mediated approach to label the SVZ-derived cells and analyze whether these labeled cells could mature into Som+ cells in the rat OB. Postnatal SVZ-derived cells were labeled by focal injections of retroviruses engineered to express enhanced green fluorescent protein (GFP) [15], [16], and we analyzed whether these GFP+ cells could matured into Som+ cells in the adult rat OB. As shown in Figure 2 D and E, the GFP+/Som+ cells were observed in the GL of the rat OB, and nearly all of these double labeled cells express Sp8 (GFP+/Som+/Sp8+, Fig. 2 D and E). Quantitative analysis revealed that there were 845±101 (n = 10) GFP+/Sp8 cells/mm^3^. This result also suggested that postnatal SVZ-derived cells could matured into Som+ cells in the rat OB, and this cells express Sp8.

**Figure 3:**
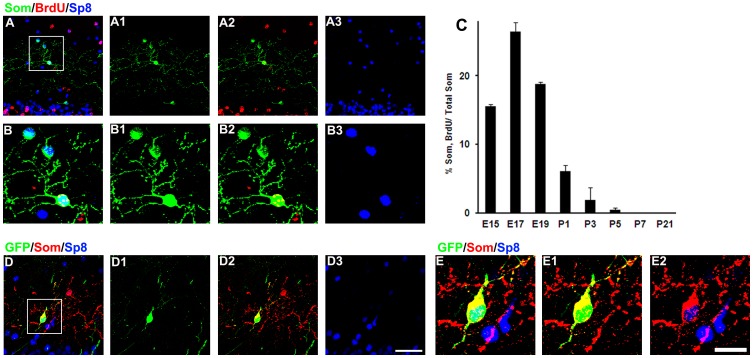
Som+ cells in the EPL of mouse OB are produced before birth and express Sp8. (A) Representative images of the BrdU+/Som+/Sp8+ cells in the EPL of the mouse OB. (B) High magnification of the boxed areas in A showing the BrdU+/Som+/Sp8+ cells. (C) Quantification of the percentage of Som+ cells that were labeled with BrdU in the EPL of the mouse OB. (D) Representative images of the GFP+/Som+/Sp8+ cells in the EPL of the mouse OB four weeks after the replication-incompetent retroviruses encoding GFP were injected into the SVZ of the P0 rat. Scale bars: 50 µm (in D3 applies to A and D), 20 µm (in E2 applies to B and E).

The temporal production of the Som+ cells in the mouse OB was also investigated by the BrdU pulse-labeling paradigm, as in the rat experiments. The production of Som+ cells in the mouse EPL is mainly confined to the late embryonic and early postnatal stages, and the Som+ cells labeled by BrdU also express Sp8 (E15 to P3, Fig. 3 A–C, BrdU+/Som+/Sp8+ cells). We rarely found any Som+ cells in the EPL after BrdU was injected into P5 mice (Fig. 2 C), indicating that the Som+ cells are not generated in the adult mice [5], [17]. As in the rat OB, the P0 mice were subjected to the focal injections of retroviruses engineered to express GFP into SVZ. We found GFP+/Som+ cells in the mouse inner EPL, and nearly all of these double label cells express Sp8 as shown in Fig. 2 D and E. To our surprise, the density of the GFP+/Som+ cells in the mouse OB was less than 200 (145±13, n = 3, Fig. 1 O-R)/mm^3^, far more less than that in the rat experiment (P<0.05, n = 3). These results were consistent with the BrdU birth dating data that Som+ interneurons in the OB are mainly produced before birth in the mouse and after birth in the rat.

### The Dlx5/6 and Emx1 Lineage Contributes to Som+ interneuron in the EPL of Mouse OB

**Figure 4:**
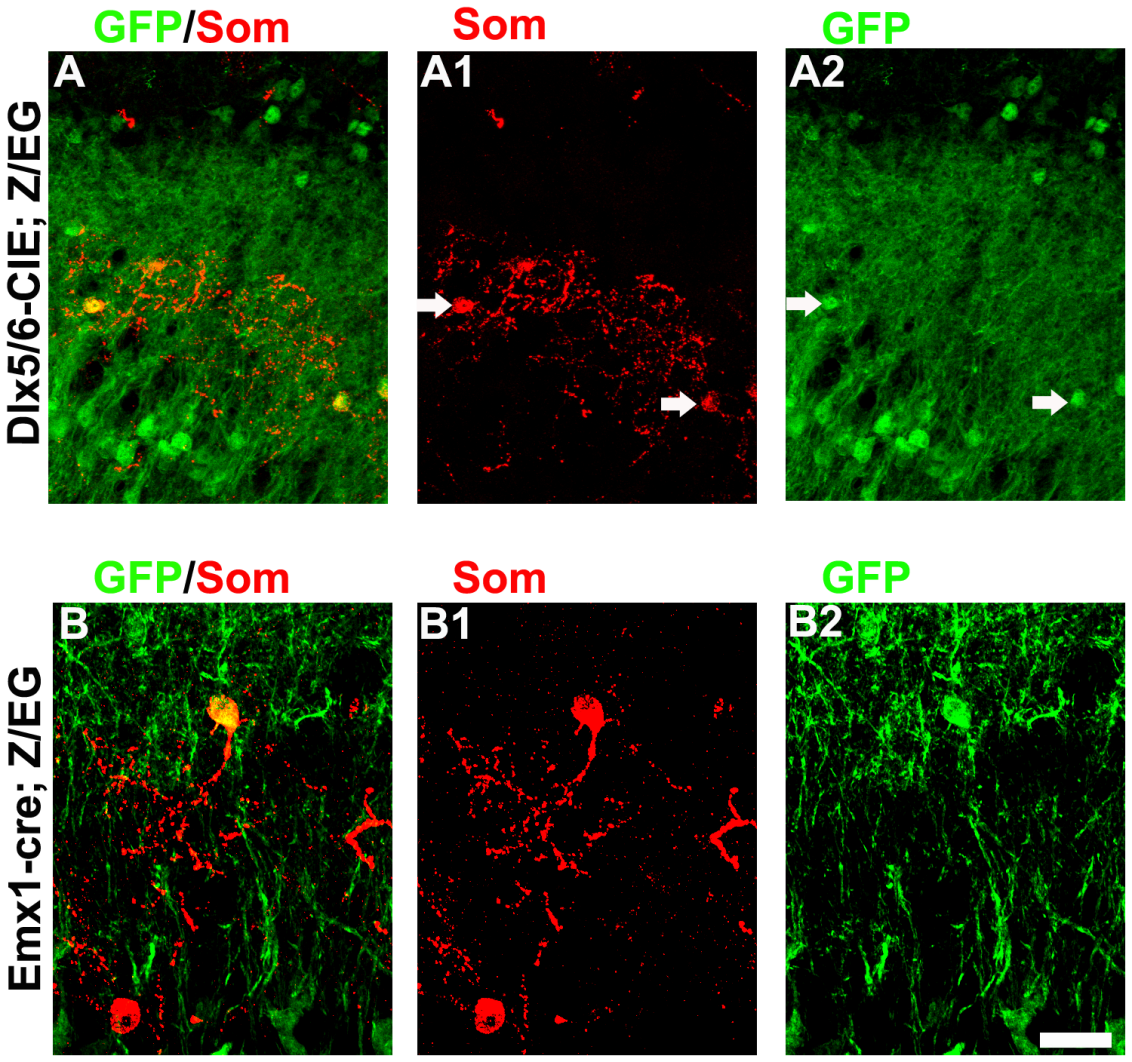
Dlx5/6 and Emx1 lineage contribute to the Som+ interneurons in the OB. (A) Representative images of the GFP+ cells that express Som in the OB of adult Dlx5/6-CIE; Z/EG mice. (B) Representative images of the GFP+ cells that express Som in the OB of adult Emx1-Cre; Z/EG mice. Scale bars: 50 µm (in B2 applies to A and B).

Previous studies have demonstrated that Dlx5/6 lineage contributes TH+, CB+, CR+ and PV+ interneurons in the OB [3], [9], [17]. We used Dlx-CIE mice to investigate the extent of Som+ cells that derived from Dlx5/6-lineage. We crossed Dlx5/6-CIE mice to Z/EG reporter mice (Dlx5/6-CIE; Z/EG), and found the GFP expression in the OB as shown previously [3], [9] (Fig. 4 A). In the EPL, we found that more that 60% of Som+ colocalized with GFP in the OB of Dlx5/6-CIE; Z/EG mice (GFP+/Som+ cells, Fig. 4 A), these GFP+/Som+ cells exhibited the same morphologies as those in the normal CD1 mice.

Emx1-expressing progenitors in the neonatal and adult brains give rise to OB interneuron subtypes that express TH, CB, and CR [3], and we found recently that some individual pallial Emx1-lineage progenitors have the potential to generate both projection neurons and interneurons in vitro [18]. However, whether Emx1-lineage contributes to Som+ cells in the OB was unknown. We crossed the Emx1-cre mouse to the Z/EG reporter mouse, that permanently expresses GFP in cells that had expressed Cre at some point during their development (Emx1-cre; Z/EG) [19]. In addition to extensive GFP-labeled cells in the GCL and GL, GFP-labeled cells were found in the EPL (Fig. 4 B). Interestingly, we found that parts of (6.53±1.8%, n = 3) the Som+ cells colocalized with the GFP in the EPL of the Emx1-cre; Z/EG mice (Fig. 4B).

### Conditional Inactivation of Sp8 Results in the Loss of Som+ interneurons in the EPL of the Mouse OB

Previous studies have demonstrated that the numbers of CR+ cells in the GCL and GL, and CB+ and TH+ in the GL, were greatly decreased by conditional inactivation of Sp8 [12]. We found that Sp8 regulated the production/or survival of the PV cells in the EPL of mouse OB [9]. In the present study, we investigated the function of Sp8 in the generation and/or survival of the Somatostatin interneurons by genetically ablated Sp8 in the Emx1, Dlx5/6 and Nestin lineage [12].

**Figure 5:**
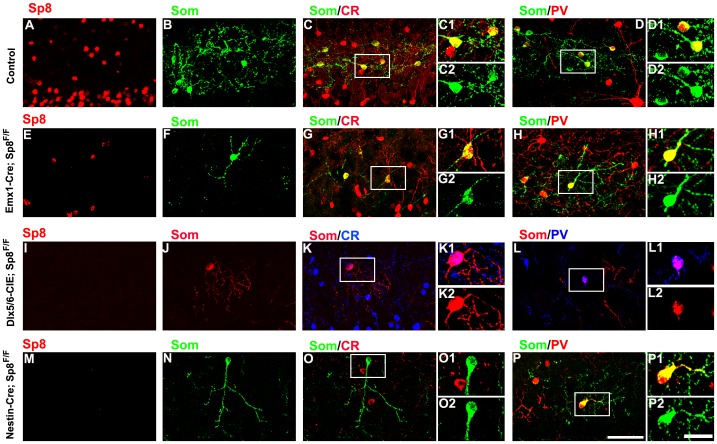
The density of the Som+ cells in the EPL of the OB is significantly decreased in the Sp8 conditional mutant mice. (A–D) The representative images that showing the expression of Sp8 (A), Som+ (B), Som+/CR+ (C) and Som+/PV+ (D) cells in the EPL in the OB of adult control mice. (E–H) The representative images showing the expression of Sp8 (E), Som+ (F), Som+/CR+ (G) and Som+/PV+ (H) cells in the EPL in the OB of adult Emx1-Cre; Sp8 ^Flox/flox^ mutant mice. (I–L) The representative images that showing the expression of Sp8 (I), Som+ (J), Som+/CR+ (K) and Som+/PV+ (L) cells in the EPL in the OB of adult Dlx5/6-CIE; Sp8 ^Flox/flox^ mutant mice. (M–P) The representative images that showing the expression of Sp8 (M), Som+ (N), Som+/CR+ (O) and Som+/PV+ (P) cells in the EPL in the OB of adult Nestin-Cre; Sp8 ^Flox/flox^ mutant mice. Scale bars: 50 µm (bar in P applies to A–P), 20 µm (in P2 applies to C1, C2, D1, D2, G1, G2, H1, H2, K1, K2, L1, K2, O1, O2, P1 and P2).

As has been reported previously, the adult conditional Sp8 mutants (Emx1-Cre; Sp8 ^Flox/flox^) develop consistently smaller olfactory bulbs than the controls [9], [12]. The SVZ and RMS were normal in the Emx1-Cre; Sp8 ^Flox/flox^ mutants. There were no ectopic Som+ cells localized in the RMS, especially at the OB level (data not shown). The EPL was reduced in size, and Sp8 expression was severely abolished in the OB of the Emx1-Cre; Sp8 ^Flox/flox^, compared to the control group (Fig. 5 A). The density of the Som+ cells in the EPL was significantly decreased in the Emx1-Cre; Sp8 ^Flox/flox^ mutant mice compared to the controls (Fig. 5 F), and the quantification data revealed that more than 60% (65±4.2%, n = 3) of the Som+ cells were reduced in the conditional mutant group (Fig. 3 L).Consistent with a previous study [7], nearly all of the Som+ interneurons express CR in the control group (Fig. 5 C), and we found that majority of the remained Som+ interneuron express CR after genetic ablation of Sp8 in the Emx1-lineage (Fig. 5 G). Interestingly, we found that less than 20% of the Som+ interneurons expressed parvalbumin (PV+/Som+ cells) (Fig. 5 H), the percentage decreased significantly compared with control group (Fig. 5 D and H, 16±1.4% in the mutant group *vs* 49±1.2% in the control group, P<0.05, n = 3) in the EPL of the Emx1-Cre; Sp8 ^Flox/flox^ mutant OB.

To further explore the role of Sp8 in the generation and/or survival of the Som+ cells, we made use of the Dlx5/6-CIE mice, which express Cre in migrating neuroblasts and differentiating olfactory bulb interneurons [9], [12], [20]. As in the Emx1-Cre; Sp8^ Flox/flox^ mutant mice, the olfactory bulb of the adult Dlx5/6-CIE; Sp8^ Flox/flox^ mutants was consistently smaller than the olfactory bulb of the controls, the SVZ and RMS were normal in the Dlx5/6-CIE; *Sp8* ^Flox/flox^ mutants. There were no ectopic Som+ cells localized in the RMS, especially at the OB level (data not shown). The size of the EPL was reduced, and Sp8 expression was completely abolished (Fig. 5I). The density of the Som+ cells in the mutant OB was significantly reduced by 80% in the Dlx5/6-CIE; Sp8^ Flox/flox^ mutants (Fig. 5I). Different from control groups that nearly all of the Som+ cells express CR, about one-third (31±1.6%, n = 3) of Som+ cells did not express CR and the CR expression in the Som+ cell decreased greatly (Fig. 5 K). Surprisingly, all of the remaining Som+ cells in the EPL expressed PV (PV+/Som+ cells, Fig. 5L).

To further investigate the role of Sp8 in the generation and/or survival of the Som+ cells, we genetically ablated Sp8 specifically in neural stem and precursor cells by taking advantage of the well-characterized nestin-cre transgene mouse line [21], [22]. As in the Dlx5/6-CIE; Sp8^ Flox/flox^ mutants, the olfactory bulb of the adult Nestin-Cre; Sp8^ Flox/flox^ mutants was consistently smaller than the olfactory bulb of the controls [9], [12]. The SVZ and RMS were normal in the Nestin-Cre; *Sp8* ^Flox/flox^ mutants. There were no ectopic Som+ cells localized in the RMS, especially at the OB level (data not shown). The size of the EPL was reduced, and Sp8 expression was completely abolished (Fig. 5N). The density of the Som+ cells in the mutant OB was reduced to nearly two percent of the controls (2±0.4%, n = 3). Surprisingly, the remaining Som+ cells that did not express CR (Fig. 5 O) and nearly all of them express PV (Fig. 5 P).

## Discussion

In this study, we demonstrated that majority of the Som+ interneurons expressed the transcription factor Sp8, and the Som+ interneurons in the OB are produced around the time of birth. The genetic ablation of Sp8 led to a significant reduction in Som+ interneurons in the EPL of the OB.

Sp8, a member of the Sp1 zinc finger transcription factor family, is expressed in the embryonic dLGE and postnatal SVZ-RMS-OB system [9], [12], 13,23. In the present study, we show that virtually all the Som+ in the OB express Sp8. These results suggest that the Som+ cells in the OB might be derived from the embryonic dLGE.

The generation of new neurons in the OB begins in the embryonic stages and continues throughout life. BrdU, an analog of thymidine, can replace thymidine and permanently incorporate into the newly synthesized DNA of dividing cells during the S-phase of the cell cycle. However, in proliferating progenitors, such as the stem or transit amplifying cells, the BrdU label is diluted in half during each round of division [24]. Thus, the BrdU pulse-labeling method used in our study is efficient for labeling the neuroblasts but not the transit-amplifying progenitors and primary neural stem cells. This trait could enable us to narrow down the birthdates of the OB interneurons to a more strict time window [6], [9], [25], [26]. Moreover, the half-life time of BrdU is approximately 4–6 hours *in vivo*. Multiple injections (at least 4 times) of BrdU were required to evaluate the total number of the newly generated neurons during 24 hours. That requirement means that a single injection of BrdU could only effectively label approximately one quarter of the newly generated neurons during 24 hours. We found that more than 6% percent of the Som+ neurons were produced during P3 in rats with a single injection of BrdU, which meant that more than one quarter of the Som+ neurons were produced during this time; this interval could be considered the generation peak. Nearly one quarter of the BrdU+/Som+ cells were detected in the present study by a single injection of BrdU, meaning that most (more that 90%) of the Som+ cells were produced during E17-P20.

Previous studies have demonstrated that Dlx5/6-lineage contribute CR+, CB+ and TH+ interneurons in the OB [3]. Nearly 30% of the olfactory bulb interneurons in the mouse EPL were derived from the Emx-1 lineage [17]. In the present study, we found that about 60% of Som+ cells in the EPL are derived from Dlx5/6 lineage, and the Emx1 lineage contributes less than 10% of the Som+ cells in the EPL of the OB in mice, suggesting that the contribution of the Dlx5/6 and Emx1 lineage is underestimated in the present study. Indeed, only approximately 60% of PV+ cells express GFP+ in the *Dlx5/6-CIE-Z/EG* mice. However, more than 80% of PV+ cells were reduced in the Dlx5/6-CIE; *Sp8 ^flox/flox^* mice. These results supported the notion that fate mapping within these reporter lines may be incomplete [9], [27], and more than 10% (less than 30%) of the Som+ cells in the EPL might be derived from the Emx1 lineage.

In the present study, we demonstrated that the density of the Sp8*+* and Som+ cells in the EPL is greatly reduced in the conditional mutation of Sp8 in the Emx1 lineage (>60%). Considering that the Emx1 lineage might contribute less than one third of the Som+ cells in the OB, other mechanisms regulated by Sp8, such as neuroblast migration and interneuron differentiation, might be involved in this process. It is possible that Sp8 may play an important role in these processes by controlling the expression of unknown downstream genes in a cell-autonomous way. Previous studies have shown clusters of neurons within the RMS, specifically at the olfactory bulb level, that express markers of differentiated interneurons such as calretinin, calbindin and GAD in the Sp8 conditional mutant. Radial glial morphology appears relatively normal in most areas of the conditional mutant olfactory bulb, suggesting that the migration defect is likely to be cell autonomous [12]. However, we did not find any ectopic Som+ cells localized in the SVZ or RMS in the Emx1-Cre; Sp8^Flox/flox^, Dlx5/6-CIE; Sp8^Flox/flox^ and the Nestin-Cre; Sp8^Flox/flox^ mutants. This result suggested that the ability of the neuroblasts to undergo interneuron differentiation and radial migration might be impaired, and a cell death program might be initiated, as large numbers of Som+ cells in the EPL were lost in the Emx1-Cre; Sp8^Flox/flox^ mutants.

The cytoarchitecture of the olfactory bulb was also severely disrupted in the Emx1-Cre; Sp8^Flox/flox^, Dlx5/6-CIE; Sp8^Flox/flox^ and Nestin-Cre; Sp8^Flox/flox^ mutants. This indicated that interneurons generated at embryonic stages play crucial role in organizing the cytoarchitectural organization of the olfactory bulb. However, we can not entirely exclude the possibility that non-cell autonomous mechanisms are involved in this process. Future studies in which Sp8 is intact embryonically and conditionally inactivated at postnatal stages may help to address this issue.

We also observed that the density of the PV+ and PV+/Som+ cells in the EPL were also affected by the conditional ablation of Sp8 in the Emx1 lineage, and differential loss of the specific interneuron subtypes might have occurred. This finding suggests that although the PV+ and the Som+ cells are both derived from the dLGE, the progenitor populations that give rise to these respective cell types may be intrinsically divergent. Indeed, previous studies that have demonstrated a mosaic distribution of progenitors have also raised the possibility that the activity of stem cells is regionally modulated to regulate the production of different types of interneurons [4].

In the present study, we found that parts of the Som+ cells in the EPL did not express CR in by conditional ablation of Sp8 in the Dlx5/6 and Nestin lineage. This observation suggests that despite the regulation of Sp8, other mechanism might be involved in the regulation of these two subtypes of interneurons. In the neocortex, CR+ cells derived from LGE/dCGE that did not express Som+ are regulated by Dlx1, whereas, the CR+/Som+ cells that derived from MGE are regulated by Lhx6 in addition to Dlx1 [28].

More Som+ cells were lost, and nearly all of these remaining Som+ cells expressed PV (PV+/Som+ cells), in the Nestin-cre; Sp8^Flox/flox^ mutants. This observation suggests that despite expressing Sp8, the genetic programs that regulate the generation or survival of the PV+ and Som+ interneurons might be different. A similar phenomenon was found in the neocortex, where the Som+ and the PV+ interneurons are differentially regulated by the same regulator. A recent study has demonstrated that the conditional removal of Satb1 in mouse interneurons results in the loss of the majority of Som+ cells, as well as some PV+ cells, in the neocortex [29].The characterization of the role of Sox6 in the PV+ and the Som+ interneurons revealed that while the removal of Sox6 resulted in the mispositioning of both the PV+ and the Som+ interneurons, it only significantly affected the survival of the PV+ interneuron population [30].

The present data demonstrate that the Som+ and the PV+ cells in the OB are the most affected interneuron subtypes in the Sp8 conditional mutant mouse. These results indicate that Sp8 is crucial for the normal development of the OB interneurons.

## Materials and Methods

### Animals

The CD-1 mice and the Wistar rats were obtained from the Shanghai SLAC Laboratory Animal Co. Ltd (Shanghai, China). Dlx5/6-CIE mice were a gift from Kenneth Campbell [12]. The Emx1-Cre (Gorski et al., 2002) mice were obtained from the Jackson Laboratory (Bar Harbor, ME, USA. Strain name: B6.129S2-Emx1^tm1(cre)Krj^/J). This strain expresses Cre recombinase from the endogenous Emx1 locus, and when crossed with a strain containing a loxP-site flanked sequence, Cre-mediated recombination results in tissue-specific deletion of the flanked sequence. The Nestin-Cre mice were obtained from the Jackson Laboratory (Strain name: B6.Cg-Tg(Nes-cre)1Kln/J), and these transgenic mice express Cre recombinase under the control of the rat nestin promoter and enhancer. Z/EG mice were obtained from the Jackson Laboratory (Strain name: STOCK Tg(CAG-Bgeo/GFP)21Lbe/J), and these Z/EG transgenic mice constitutively express lacZ under the control of the CMV enhancer/chicken actin promoter. When this strain was crossed with a Cre recombinase-expressing strain, lacZ expression is replaced with enhanced GFP expression in tissues expressing cre. The Emx1-Cre, the Nestin-Cre and the Sp8 ^Flox/flox^ mice were genotyped as previously described [12]. The Sp8 conditional mutant mice were obtained from crossing double heterozygous males (Emx1-Cre; Sp8 ^Flox/+^, Dlx5/6-CIE; Sp8^Flox/+^ or Nestin-Cre; Sp8^ Flox/+^) with Sp8 homozygous flox (Sp8 ^Flox/flox^) females (Waclaw et al., 2006). The heterozygote or wild type mice were used as the controls. The experiments were carried out on adult mice. All of the experiments using animals were carried out in accordance with the National Institutes of Health’s Guide for the Care and Use of Laboratory Animals revised in 1996, and the study was approved by the Fudan University Animal Care and Use Committee. Every effort was made to minimize the number of animals used.

### BrdU Injections

In the present study, the BrdU pulse-labeling method was used to pulse-label the newly born neurons at each embryonic time point. In this method, mice and/or rats were given a single BrdU injection at different developmental stages, and the BrdU-labeled nuclei in the OB were quantified after different survival times. This method is widely used to determine the quantity of newly generated cells that are preferentially produced at different ages. Using the BrdU labeling technique may enable us to narrow down the birthdates of the OB interneurons to a more strict time window [6], [9], [25], [26]. BrdU (100 mg/kg body weight; Sigma, St. Louis, Mo, USA) [6], [9], [13], [26] was administered once to pregnant rodent mothers via intraperitoneal injection at E15, E17 and E19 for the CD1 mice and at E17 and E19 for the Wistar rats. The animals were killed 6–7 weeks after the BrdU injections. After birth, the BrdU (100 mg/kg) was injected intraperitoneally once to the postnatal mice or rats at postnatal day 1 (P1), P3, P5, P7, P21 and P60. The animals were killed 6–7 weeks after the BrdU injections.

### Retrovirus Injections

Engineered self-inactivating murine oncoretroviruses were used to express GFP specifically in proliferating cells [15], [16]. High titers of the engineered retroviruses (1×10^9^ unit/ml) were produced by cotransfecting retroviral vectors and VSVG into 293 gp cells followed by ultracentrifugation of the viral supernatant, as previously described [16], [27]. The P0 CD1 mice or Wistar rats were anesthetized, and 1 µl of retroviruses was stereotaxically injected into the lateral ventricles with the following coordinates: anterior = 0.3 mm from the bregma, lateral = ±1.2 mm, ventral = 1.7 mm for the rats, and 0 mm from the bregma, lateral = ±0.8 mm, ventral = 1.4 mm for the mice. The animals were euthanized 4 weeks after the retroviral injection. All of the animal care was in accordance with the institutional guidelines.

### Immunohistochemistry

The mice and rats were deeply anesthetized before intracardiac perfusion with 4% paraformaldehyde. The brains were post-fixed with 4% paraformaldehyde overnight and then cryoprotected at least 24 hours in 30% sucrose in 0.1 M phosphate buffer (pH 7.4). The brain samples were frozen in embedding medium (O.C.T.; Sakura Finetec, Torrance, CA) on a dry ice/ethanol slush.

Free-floating coronal sections of the OB were collected in 30 μm thickness in 6-well plates and were sampled 180 μm apart. The sections for BrdU staining were pretreated with 2 N HCl for 1 hour at room temperature to denature the DNA. The sections were then blocked for 1 hour in Tris-buffered saline (TBS; pH 7.4) with 10% donkey serum and 0.5% Triton X-100. The primary antibodies were applied for overnight incubation at 4°C. The following antibodies were used: anti-BrdU (rat monoclonal, 1: 30, Accurate Chemical, Westbury, NY, USA), anti- neuronal nuclei (mouse monoclonal, 1: 400, Chemicon, Temecula, CA, USA), anti-somatostatin (rabbit polyclonal, 1∶100, Santa Cruz Biotechnology, CA, USA), anti-Sp8 (goat polyclonal, 1∶500, Santa Cruz Biotechnology, CA, USA), anti-GFP (chicken monoclonal, 1∶2000, Aves Labs, Tigard, OR, USA), anti-parvalbumin (mouse monoclonal, 1∶400, Chemicon, Temecula, CA, USA), and anti-calretinin (mouse monoclonal, 1∶1,000, Swant, Bellinzona, Switzerland). The secondary antibodies against the appropriate species were incubated for 2 h at room temperature (1: 200, all from Jackson, Bar Harbor, ME, USA). All the secondary antibody combinations were carefully examined to ensure that there was no cross-talk between the fluorescent dyes or cross-reactivity between the secondary antibodies, especially for the anti-rat and the anti-mouse secondary antibodies. DAPI (Sigma, 1 µg/ml) was used to counterstain the nuclei. The fluorescently stained sections were coverslipped with Gel/Mount (Biomeda, Foster City, CA, USA). Streptavidin and diaminobenzidine (DAB) were used to visualize the reaction product for the bright-field staining sections. The omission of primary antibodies eliminated the staining.

### Microscopy and Cell Quantification

The fluorescently immunolabeled sections were analyzed on an Olympus FV1000 confocal laser scanning microscope. The confocal Z sectioning was performed at 1 μm intervals using a 40 × (NA = 1.0) objective, or 0.5 μm intervals using a 60 × (NA = 1.42) objective. The orthogonal images were examined to confirm the co-localization. The images were acquired, and a Z-stack was reconstructed using FV10-ASW software, cropped, adjusted, and optimized in Adobe Photoshop 9.0 (Adobe Systems Inc, San Jose, CA, USA).

To quantify the cells in the section, 10 non-overlapping fields (200 µm× 200 µm) from each 30 µm section at 180 µm intervals were analyzed using an Olympus FV1000 with a 60 × objective (NA = 1.42); from each OB, six coronal sections were quantified (*n = *3–5 animals per group). This method allowed us to accurately count the number of double-labeled cells in the OB [6], [9], [31]. The density of the double-labeled cells in the OB was obtained by dividing the total volume analyzed by the total number of cells counted. For example, the total volume analyzed per OB was 10 fields ×6 sections×30 µm section thickness × 200 µm × 200 µm = 0.072 mm^3^. The density of the double-labeled cells per OB was obtained by dividing 0.072 by the total number of cells counted.

All the data are presented as the mean±SEM and were analyzed for statistical significance using Student’s *t*-test. We considered *p* values <0.05 statistically significant.

## Supporting Information

Figure S1 **Som+ cells in the GL of rat OB do not express CR, TH, or CB.** (A–C), Som+ cells in the GL of rat OB do not express CR. A1’ and A1” are high-magnification images of boxed areas in A. (D–F), Som+ cells in the GL of rat OB do not express TH. D1’ and D1” are high-magnification images of boxed areas in D. (G–I), Som+ cells in the GL of rat OB do not express CB. G1’ and G1” are high-magnification images of boxed areas in G. Scale bars: 100 µm (in I applies to A and I), 20 µm (in G1” applies to A1’–G1”).
(TIF)

Figure S2 **The Som+ cells in the GL of rat OB express Sp8.** (A), lower magnification of images showing the Som+ cells in the GL of rat OB express Sp8. (B and C), Orthogonal views of the boxed areas in A showing the Som+/Sp8+ cells. (B1–B4 and C1–C4), Four consecutive 0.5 µm confocal merged images showing Som and Sp8 immunostaining, respectively. Scale bars: 100 µm (in A), 20 µm (in C applies to B and C).
(TIF)

## References

[1] Alvarez-Buylla A, Lim DA (2004) For the long run: Maintaining germinal niches in the adult brain. Neuron 41: 683–686. 15003168 10.1016/s0896-6273(04)00111-4

[2] Ming GL, Song H (2005) Adult neurogenesis in the mammalian central nervous system. Annu Rev Neurosci 28: 223–250. 16022595 10.1146/annurev.neuro.28.051804.101459

[3] Kohwi M, Petryniak MA, Long JE, Ekker M, Obata K, et al (2007) A subpopulation of olfactory bulb GABAergic interneurons is derived from Emx1- and Dlx5/6-expressing progenitors. J Neurosci 27: 6878–6891. 17596436 10.1523/JNEUROSCI.0254-07.2007 PMC4917362

[4] Merkle FT, Mirzadeh Z, Alvarez-Buylla A (2007) Mosaic organization of neural stem cells in the adult brain. Science 317: 381–384. 17615304 10.1126/science.1144914

[5] Batista-Brito R, Close J, Machold R, Fishell G (2008) The distinct temporal origins of olfactory bulb interneuron subtypes. J Neurosci 28: 3966–3975. 18400896 10.1523/JNEUROSCI.5625-07.2008 PMC2505353

[6] Yang Z (2008) Postnatal subventricular zone progenitors give rise not only to granular and periglomerular interneurons but also to interneurons in the external plexiform layer of the rat olfactory bulb. J Comp Neurol 506: 347–358. 18022946 10.1002/cne.21557

[7] Lepousez G, Csaba Z, Bernard V, Loudes C, Videau C, et al (2010) Somatostatin interneurons delineate the inner part of the external plexiform layer in the mouse main olfactory bulb. J Comp Neurol 518: 1976–1994. 20394054 10.1002/cne.22317

[8] Gutierrez-Mecinas M, Crespo C, Blasco-Ibanez JM, Gracia-Llanes FJ, Marques-Mari AI, et al (2005) Characterization of somatostatin- and cholecystokinin-immunoreactive periglomerular cells in the rat olfactory bulb. J Comp Neurol 489: 467–479. 16025459 10.1002/cne.20649

[9] Li X, Sun C, Lin C, Ma T, Madhavan MC, et al (2011) The transcription factor sp8 is required for the production of parvalbumin-expressing interneurons in the olfactory bulb. J Neurosci 31: 8450–8455. 21653849 10.1523/JNEUROSCI.0939-11.2011 PMC3124650

[10] Ninkovic J, Pinto L, Petricca S, Lepier A, Sun J, et al (2010) The transcription factor Pax6 regulates survival of dopaminergic olfactory bulb neurons via crystallin alphaA. Neuron 68: 682–694. 21092858 10.1016/j.neuron.2010.09.030 PMC4388427

[11] Kohwi M, Osumi N, Rubenstein JLR, Alvarez-Buylla A (2005) Pax6 is required for making specific subpopulations of granule and periglomerular neurons in the olfactory bulb. Journal of Neuroscience 25: 6997–7003. 16049175 10.1523/JNEUROSCI.1435-05.2005 PMC6724841

[12] Waclaw RR, Allen ZJ, 2nd, Bell SM, Erdelyi F, Szabo G, et al (2006) The zinc finger transcription factor Sp8 regulates the generation and diversity of olfactory bulb interneurons. Neuron 49: 503–516. 16476661 10.1016/j.neuron.2006.01.018

[13] Liu F, You Y, Li X, Ma T, Nie Y, et al (2009) Brain injury does not alter the intrinsic differentiation potential of adult neuroblasts. J Neurosci 29: 5075–5087. 19386903 10.1523/JNEUROSCI.0201-09.2009 PMC6665479

[14] Kosaka K, Kosaka T (2007) Chemical properties of type 1 and type 2 periglomerular cells in the mouse olfactory bulb are different from those in the rat olfactory bulb. Brain Res 1167: 42–55. 17662264 10.1016/j.brainres.2007.04.087

[15] Duan X, Chang JH, Ge S, Faulkner RL, Kim JY, et al (2007) Disrupted-In-Schizophrenia 1 regulates integration of newly generated neurons in the adult brain. Cell 130: 1146–1158. 17825401 10.1016/j.cell.2007.07.010 PMC2002573

[16] van Praag H, Schinder AF, Christie BR, Toni N, Palmer TD, et al (2002) Functional neurogenesis in the adult hippocampus. Nature 415: 1030–1034. 11875571 10.1038/4151030a PMC9284568

[17] Young KM, Fogarty M, Kessaris N, Richardson WD (2007) Subventricular zone stem cells are heterogeneous with respect to their embryonic origins and neurogenic fates in the adult olfactory bulb. J Neurosci 27: 8286–8296. 17670975 10.1523/JNEUROSCI.0476-07.2007 PMC6331046

[18] Cai Y, Zhang Y, Shen Q, Rubenstein JL, Yang Z (2013) A subpopulation of individual neural progenitors in the Mammalian dorsal pallium generates both projection neurons and interneurons in vitro. Stem Cells 31: 1193–1201. 23417928 10.1002/stem.1363

[19] Novak A, Guo C, Yang W, Nagy A, Lobe CG (2000) Z/EG, a double reporter mouse line that expresses enhanced green fluorescent protein upon Cre-mediated excision. Genesis 28: 147–155. 11105057

[20] Stenman J, Toresson H, Campbell K (2003) Identification of two distinct progenitor populations in the lateral ganglionic eminence: implications for striatal and olfactory bulb neurogenesis. J Neurosci 23: 167–174. 12514213 10.1523/JNEUROSCI.23-01-00167.2003 PMC6742158

[21] Isaka F, Ishibashi M, Taki W, Hashimoto N, Nakanishi S, et al (1999) Ectopic expression of the bHLH gene Math1 disturbs neural development. Eur J Neurosci 11: 2582–2588. 10383648 10.1046/j.1460-9568.1999.00699.x

[22] Vernay B, Koch M, Vaccarino F, Briscoe J, Simeone A, et al (2005) Otx2 regulates subtype specification and neurogenesis in the midbrain. J Neurosci 25: 4856–4867. 15888661 10.1523/JNEUROSCI.5158-04.2005 PMC6724764

[23] Wei B, Nie Y, Li X, Wang C, Ma T, et al (2011) Emx1-expressing neural stem cells in the subventricular zone give rise to new interneurons in the ischemic injured striatum. Eur J Neurosci 33: 819–830. 21219481 10.1111/j.1460-9568.2010.07570.x

[24] van Praag HM (2002) Why has the antidepressant era not shown a significant drop in suicide rates? Crisis 23: 77–82. 12500893 10.1027//0227-5910.23.2.77

[25] Lledo PM, Merkle FT, Alvarez-Buylla A (2008) Origin and function of olfactory bulb interneuron diversity. Trends Neurosci 31: 392–400. 18603310 10.1016/j.tins.2008.05.006 PMC4059175

[26] Lemasson M, Saghatelyan A, Olivo-Marin JC, Lledo PM (2005) Neonatal and adult neurogenesis provide two distinct populations of newborn neurons to the mouse olfactory bulb. J Neurosci 25: 6816–6825. 16033891 10.1523/JNEUROSCI.1114-05.2005 PMC6725349

[27] e S, Goh EL, Sailor KA, Kitabatake Y, Ming GL, et al (2006) GABA regulates synaptic integration of newly generated neurons in the adult brain. Nature 439: 589–593. 16341203 10.1038/nature04404 PMC1420640

[28] {Wang W, Y., Dye CA, Sohal V, Long JE, Estrada RC, et al (2010) Dlx5 and Dlx6 regulate the development of parvalbumin-expressing cortical interneurons. J Neurosci 30: 5334–5345. 20392955 10.1523/JNEUROSCI.5963-09.2010 PMC2919857

[29] Close J, Xu H, De Marco Garcia N, Batista-Brito R, Rossignol E, et al (2012) Satb1 is an activity-modulated transcription factor required for the terminal differentiation and connectivity of medial ganglionic eminence-derived cortical interneurons. J Neurosci 32: 17690–17705. 23223290 10.1523/JNEUROSCI.3583-12.2012 PMC3654406

[30] Batista-Brito R, Rossignol E, Hjerling-Leffler J, Denaxa M, Wegner M, et al (2009) The cell-intrinsic requirement of Sox6 for cortical interneuron development. Neuron 63: 466–481. 19709629 10.1016/j.neuron.2009.08.005 PMC2773208

[31] Yang Z, Levison SW (2007) Perinatal hypoxic/ischemic brain injury induces persistent production of striatal neurons from subventricular zone progenitors. Dev Neurosci 29: 331–340. 17762201 10.1159/000105474

