# Supplementary figures and images for: The Production of Somatostatin Interneurons in the Olfactory Bulb Is Regulated by the Transcription Factor Sp8
Source: PLoS One. 2013 Jul 23;8(7):e70049. doi: 10.1371/journal.pone.0070049 (PMC3720950; doi:10.1371/journal.pone.0070049)

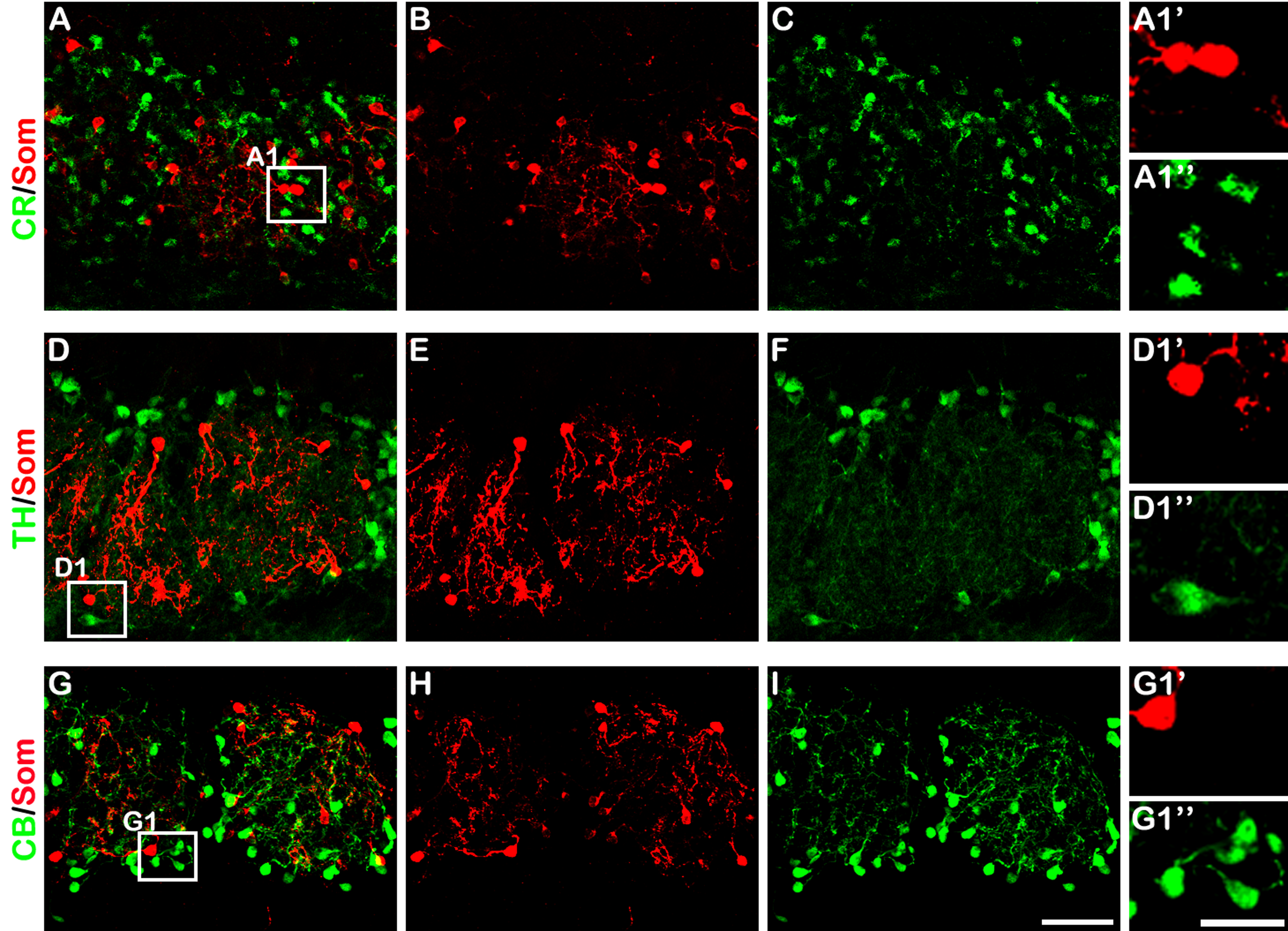

Supplement: Figure S1 — Som+ cells in the GL of rat OB do not express CR, TH, or CB. (A–C), Som+ cells in the GL of rat OB do not express CR. A1’ and A1” are high-magnification images of boxed areas in A. (D–F), Som+ cells in the GL of rat OB do not express TH. D1’ and D1” are high-magnification images of boxed areas in D. (G–I), Som+ cells in the GL of rat OB do not express CB. G1’ and G1” are high-magnification images of boxed areas in G. Scale bars: 100 µm (in I applies to A and I), 20 µm (in G1” applies to A1’–G1”). (TIF) [file pone.0070049.s001.tif]

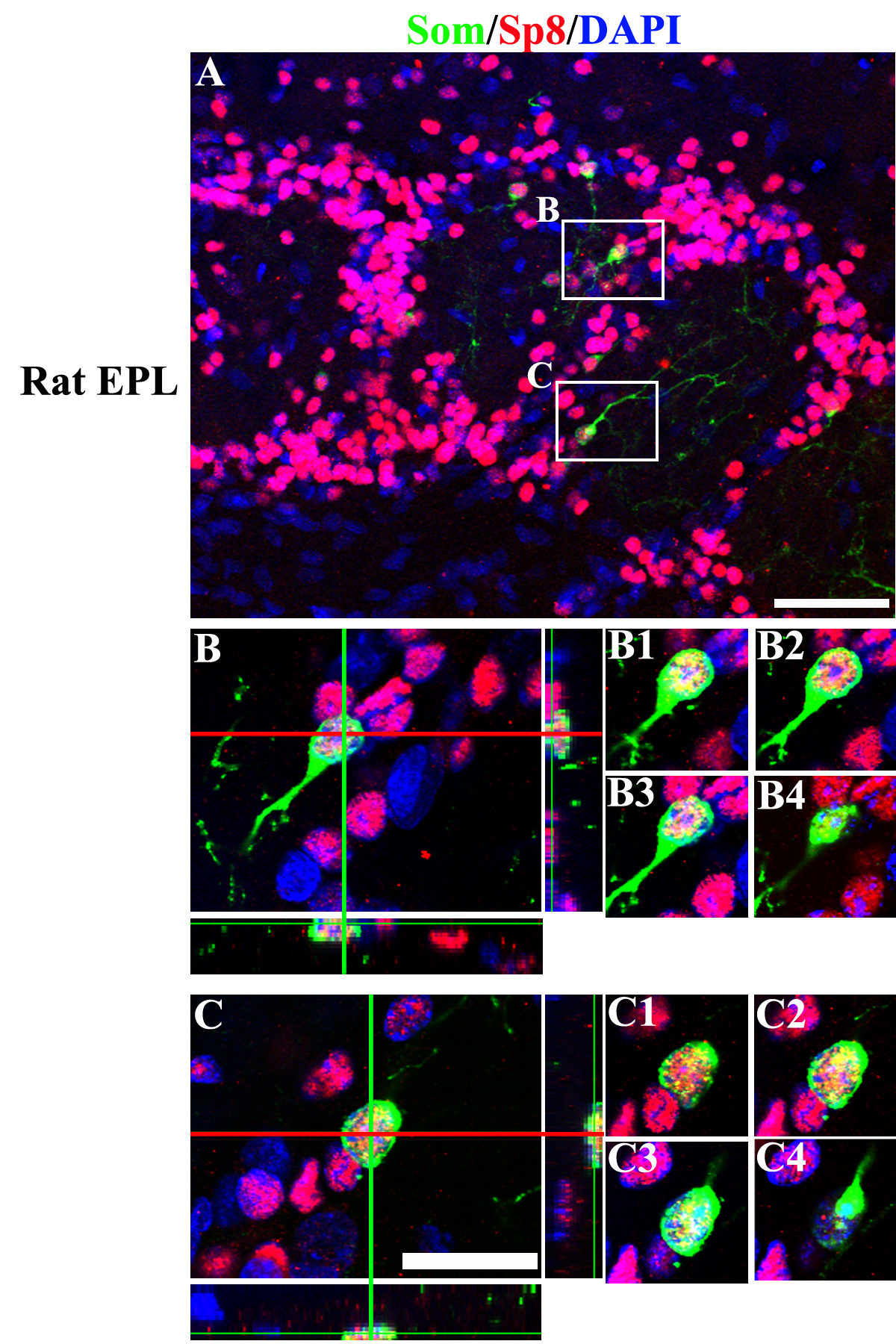

Supplement: Figure S2 — The Som+ cells in the GL of rat OB express Sp8. (A), lower magnification of images showing the Som+ cells in the GL of rat OB express Sp8. (B and C), Orthogonal views of the boxed areas in A showing the Som+/Sp8+ cells. (B1–B4 and C1–C4), Four consecutive 0.5 µm confocal merged images showing Som and Sp8 immunostaining, respectively. Scale bars: 100 µm (in A), 20 µm (in C applies to B and C). (TIF) [file pone.0070049.s002.tif]
